# Immobilization of heavy metals by microbially induced carbonate precipitation using hydrocarbon-degrading ureolytic bacteria

**DOI:** 10.1016/j.btre.2022.e00747

**Published:** 2022-06-09

**Authors:** Zulfa Al Disi, Essam Attia, Mohammad I. Ahmad, Nabil Zouari

**Affiliations:** a Environmental Science Program, Department of Biological and Environmental Sciences, College of Arts and Sciences, Qatar University, PO. Box 2713, Doha, Qatar; b Central Laboratory Unit, Qatar University, P. O. Box 2713, Doha, Qatar

**Keywords:** Pollution, Heavy metals, Ureolytic bacteria, Biomineralization, MICP

## Abstract

Crude oil contamination introduces multiple threats to human health and the environment, most of which are from toxic heavy metals. Heavy metals cause significant threats because of their persistence, toxicity, and bio-accumulation. Biomineralization, performed through many microbial processes, can lead to the immobilization of heavy metals in formed minerals. The potential of the microbially carbonate-induced precipitation (MICP) in removal by biomineralization of several heavy metals was investigated. A collection of diverse 11 bacterial strains exhibited ureolytic activity and tolerance to heavy metals when growing in Luria-Bertani (LB) and urea medium. Determination of the minimum inhibitory concentrations (MIC) revealed that heavy metal toxicity was arranged as Cd > Ni > Cr > Cu > Zn. Three hydrocarbon-degrading bacterial strains (two of *Pseudomonas aeruginosa* and one of *Providencia rettgeri*) exhibited the highest tolerance (MIC > 5 mM) to Cu, Cr, Zn, and Ni, whereas Cd exerted significantly higher toxicity with MIC <1 mM. At all MICP conditions, different proportions of calcium carbonate (calcite) and calcium phosphate (brushite) were formed. *Pseudomonas aeruginosa* strains (QZ5 and QZ9) exhibited the highest removal efficiency of Cr (100%), whereas *Providencia rettgeri* strain (QZ2) showed 100% removal of Zn. Heavy metal complexes were found as well. Cd removal was evidenced by the formation of cadmium phosphate induced by *Providencia rettgeri* bacterial activity. Our study confirmed that hydrocarbon-degrading ureolytic bacteria not only can tolerate heavy metal toxicity but also have the capability to co-precipitate heavy metals. These findings indicate an effective and novel biological approach to bioremediate petroleum hydrocarbons and immobilize multiple heavy metals with mineral formation. This is of high importance for ecological restoration via stabilization of soil and alleviation of heavy metal toxicity.

## Highlights

• Heavy metal toxicity to hydrocarbon-degrading ureolytic bacteria is Cd > Ni > Cr > Cu > Zn.
• The ureolytic bacteria can tolerate heavy metals and co-precipitate heavy metals.
• The precipitated minerals shifted between calcite and brushite depending on the heavy metal.
• The immobilization of heavy metals resulted in removal efficiency reaching 100%.

## 1 Introduction

Pollution caused by crude oil components constitutes a serious issue [1]. Although heavy metals are economically important for industrial applications, they cause significant threats to the environment and all living organisms [2], [3], [4], [5], [6]. However. Advancements in biotechnology have led to substantial developments in bioremediation approaches [7], [8], [9], [10], such as using microorganisms to remove heavy metals from the environment or at alleviating their toxicity [11].

Many organic compounds can be biodegraded through different biological pathways depending on the type of pollutants and treatment conditions. Heavy metals can be transformed into various forms through redox processes [12]. Some microorganisms possess the ability to accumulate heavy metals by metabolism-dependent uptake [13] and this can also be carried out intracellularly [14]. Biotreatment of heavy metals may be performed directly through surface binding of the metal to extracellular substances or cell walls [15]. It also can be indirect via heavy metal uptake through metabolic processes [16].

Biomineralization is performed through many microbial processes [17]. Biominerals can be formed from several metal ions, including Ca, Mg, Fe, and Mn, that bind to appropriate anions such as carbonate, phosphate, and oxalate [18]. Calcium carbonates and phosphates constitute are primary biominerals and biominerals are essential components of biogeochemical cycles [19, 20]. The precipitation of carbonate minerals—a central aspect of biomineralization—has been investigated extensively because of its broad range of technological applications [21]. As compared to inorganically produced minerals, biominerals are characterized with particular properties such as unique size, crystallinity, and isotopic and trace element composition [22]. Microorganisms can generate carbonate compounds (CO_3_^2−^) that react with calcium ions (Ca^2+^) to produce CaCO_3_ precipitates through microbially carbonate-induced precipitation (MICP). Similarly, heavy metals with an ionic radius similar to Ca^2+^, such as Pb^2+^ and Hg^2+^, can replace Ca^2+^ ions and be incorporated into the CaCO_3_ crystal lattice [23]. In this way, heavy metals can be immobilized in contaminated soils [24, 25]. Several other MICP applications have been reported, such as CO_2_ capture, oil recovery, self-healing of concrete structures, and soil stabilization [26], [27], [28].

Qatar is in the Arabian Gulf region, characterized by harsh climate conditions and intense weathering processes. These weathering processes cause changes in the pollutants’ structure, bioavailability, toxicity, and interactions with microbial cells [29]. Consequently, microorganisms may acquire several adaptations that increase the bioavailability of the pollutants [30]. Bacterial strains that are highly adapted to the harsh conditions (hot and dry weather) and soil (calcareous and alkaline) represent interesting models to examine how to overcome failures of bioremediation and biomineralization applications [31]. Moreover, the diversity of bacteria and their modes of adaptation to harsh conditions can lead to interference in MICP [32]. If MICP is implemented, the bioaugmented bacterial strain should be selected based on its potential for biomineralization.

In this study, the potential of a pre-selected collection of 11 diverse ureolytic bacterial strains isolated from Qatari soils was investigated. The selected bacterial strains were from the hydrocarbon-degrading or mineral-forming bacteria via MICP collections. Their tolerance to the toxicity of heavy metals and simultaneous removal of heavy metals within the formed mineral were demonstrated. The metal-biomineral complexes were further investigated to provide insight into the different mechanisms adopted by ureolytic bacteria for the removal of heaving metals from the surrounding microenvironment.

## 2 Material and methods

### 2.1 Bacterial strains

A collection of 11 diverse bacterial strains were used for this study (Table 1S, Supplementary Material). These strains were isolated in our laboratory from different soils in Qatar. The strains were shown to be either hydrocarbon-degrading or mineral-forming bacteria (Al [[29], [30], [31], [33], [34], [35]]). These strains were preserved at -80 °C in 30% glycerol. The strains were recovered by streaking on Luria-Bertani (LB) plates before each experiment to obtain fresh, pure, and viable cells.

**Table 1:** Proportions of minerals formed in the UM cultures with and without added heavy metals (semi-quantitatively assessed using MATCH software).

Strain/Medium	*Providencia rettgeri* (AZ2)	*Pseudomonas aeruginosa* (QD5)	*Pseudomonas aeruginosa* (QZ9)
**UM**	Calcite 92%, Brushite 8%	Calcite 88%, Brushite 12%	Calcite 82%, Brushite 18%
**UM-Cu**	Brushite 95%, calcite 5%,	Brushite 62%, calcite 38%	Brushite 94%, calcite 4%,
**UM-Cr**	Chromium oxide 30%, calcite 77%	Chromium oxide 77%, Calcite 33%,	Chromium oxide 66%, calcite 34%
**UM-Ni**	Brushite 83%, Calcite 17%	Brushite 99%, Calcite 1%	Brushite 61%, Calcite 39%
**UM-Cd**	Calcite 70%, Cadmium phosphate 30%	Calcite 89%, Brushite 11%	Calcite 77% Brushite 23%,
**UM-Zn**	Calcite 77%, Brushite 23%,	Calcite 97%, Brushite 3%	Calcite 95%, Brushite 5%,

### 2.2 Determination of urease activity

20 mL of urea media UM (as described in section 2.3) were inoculated with an initial inoculum of each bacterial strain with an optical density (OD_600_ nm) of 0.15. OD_600_ measurements were performed using a Lambda 25 UV/VIS spectrophotometer.  The UM cultures were incubated at 30 °C in a shaker set at 200 rpm. The bacterial strains reached their highest growth and maximum urease activity after 3 days of incubation (data not shown). At the end incubation period, each culture were centrifuged for 10 min at 14,000 rpm and the supernatant were used for determination of ureolytic activity.

The hypochlorite assay [31], with modifications, was applied to determine the urease activity by measuring ammonia in solution. The crude enzyme in the supernatant of the liquid culture from each isolate was obtained after centrifugation at 5,000 x g for 10 min. For measurement of urease activity, 100 μl of the crude enzyme was added to 900 μl of urease buffer containing HEPES 50 mM, EDTA 1 mM, and 20 g/L filter-sterilized urea with a pH of 7.5. The mixture was incubated at 25 °C for 10 min, then 200 μl of the enzyme/urea buffer solution was rapidly transferred to 400 μl of phenol-nitroprusside solution: (70 g/L phenol and 0.34 mg nitroprusside dissolved in Milli-Q water and stored in a dark bottle at 4 °C). The reaction was terminated by the addition of 400 μl of freshly prepared buffered sodium hypochlorite (NaClO) solution. The sodium hypochlorite solution contained: 75 g/L NaOH, 59 g/L Na_2_HPO_4_, 200 mL of bleach 5%, with final pH adjusted to 10.2; stored in a dark bottle at 25 °C). The sodium hypochlorite solution was made fresh daily. After 30 min of incubation at room temperature, the absorption was measured at 640 nm. Urease activity is reported as arbitrary urease activity (AUA) which is the amount of urease responsible for generating 1 μmol per minute of (NH_4_^+^) at the experimental conditions (10 min incubation at 30°C). Standard curves were established using standards prepared using serial dilutions from a 0.01 M ammonium chloride (NH_4_Cl) stock solution. The standards were run at the same time the assays were performed. The cultures and the urease activity were carried out in triplicate for each strain.

### 2.3 Tolerance of the bacterial strains to heavy metals

The toxicity of each heavy metal was studied using two culture media: Luria-Bertani (LB) and urea medium (UM). LB was composed of (g/L): NaCl 5, tryptone 10, and yeast extract 5. UM was composed of (g**/**L): NaCl 5.0, peptone 0.2, glucose 1.0, KH_2_PO_4,_ 2.0, and urea 20 (filter sterilized). Solid media were obtained by adding agar (30 g/L) to the liquid medium. All media were sterilized by autoclaving at 121°C for 20 min. The culture media were supplemented with escalating concentrations (0–10 mM) of heavy metal salts (chromium chloride, copper sulfate, nickel chloride, zinc chloride, and cadmium chloride), used separately. The culture media were inoculated with each studied strain with an initial OD_600_ of 0.15 using an inoculum prepared by incubation of 20 mL liquid medium by one separate and overnight old colony. The inoculum obtained after overnight incubation at 30 °C, has an OD_600_ of ∼3. The incubation of the cultures was for 72 h at 30 °C with continuous agitation of 150 rpm. The minimum inhibitory concentration (MIC) was expressed as the corresponding concentration causing >90% reduction in growth measured by optical density [36]. Each heavy metal concentration was tested in triplicate.

### 2.4 Determination of heavy metals removal by inductively coupled plasma optical emission

The analysis of the recovered minerals was carried out using inductively coupled plasma optical emission spectroscopy (ICP-OES; PerkinElmer Optima 7300 DV-Germany). The acid digestion followed by ICP-OES was used to determine the concentrations of heavy metals in the precipitates. The strong oxidizing acids used in the digestions process were HCl, HNO_3_, and HF added as follows: 6 mL of concentrated HCl, 2 mL of concentrated HNO_3_, and 2 mL of concentrated HF. The microwave digestion system MARS 6 (CEM Corporation) was used for sample digestion.

### 2.5 Investigation of mineral formation in the presence of heavy metal

The UM cultures (25 mL) supplemented with 0.1 M of CaCl_2_ were used to study mineral formation. Each culture was supplemented with 1 mM of Cr or Cu or Zn or Ni or 0.1 mM of Cd. The cultures were incubated for 5 days in a shaker set at 200 rpm and 30 °C. Then, the formed precipitates were recovered by centrifugation at 5000 rpm for 15 min. The supernatants were transferred to fresh falcon tubes. The pellets, including precipitated minerals and cells, were washed three times with distilled water to remove any residual and extra salts. The precipitates were air-dried at 40 °C and stored at 4 °C until further analysis. The pellets, supernatants, and washing solutions were subject to ICP-OES analysis.

### 2.6 Scan electron microscopy equipped by energy-dispersive X-ray spectroscopy and X-ray diffraction (XRD) analysis

The recovered minerals as described in section 2.5 were subjected to SEM/EDS and XRD analysis. The SEM was performed using Nova Nano Scanning Electron Microscopy equipped with Bruker EDX Detector with 5 nm resolution and a magnification of 200,000 × . The EDS was obtained following “ASTM standard method E1508–12a”, with spot size 5 at an accelerating voltage of 20 kV and 4% error.

The bulk mineralogical composition of the recovered minerals was determined using a PANalytical- multipurpose Empyrean X-ray diffractometer. Crystal Impact MATCH! Version 3.12 Software was used for the analysis of XRD spectra and semi-quantitively determination of amounts of formed minerals in each mixture.

### 2.7 Growth evaluation by determination of colony forming units

The viable number of bacterial cells in the liquid cultures was determined using the colony-forming units (CFU) method [31]. CFUs were measured by calculating the number of separate colonies formed on LB plates on which 100 µL of serial dilutions were spread and incubated overnight at 30°C.

### 2.8 Statistical analysis

Analysis of Variance (ANOVA) was conducted and significance was determined at the 95% confidence level using IBM SPSS Statistics software-Version 28.0.1.0 (142). Principal Component analysis (PCA) was performed using Origin Lab -Version 2015.

## 3 Results

### 3.1 Determination of the urease activity

Bacterial cell growth and urease activity were determined in the cultures using 20 g L^−1^ urea after 3 days of incubation as reported by Bibi *et al.* [31] (Table 2S- Supplementary Material). The bacterial growth varied between 85 ± 4 *10^6^ CFU/mL of *Pseudomonas aeruginosa* (QD5) and 17 ± 1*10^6^ CFU/mL of *Bacillus sp*. (QD1). The highest arbitrary urease activity 23.1 ± 11 AUA/ml was recorded by *Providencia rettgeri* (QZ2), while the lowest arbitrary urease activity of 6.5 ± 3 AUA/ml was recorded by *Bacillus sp*. (QD1). The highest specific activity (7.7 ± 0.6 AUA/10^7^ CFU) was obtained by *Providencia rettgeri* (QZ2) with lowest urease specific activity (1.9 ± 0.2 AUA/10^7^ CFU) by *Pseudomonas aeruginosa* (QD5). ANOVA confirmed significant variations in growth profiles among the studied isolates, as well as in their specific production of urease (P-value < 0.001).

### 3.2 Tolerance to heavy metals

**Fig. 1:**
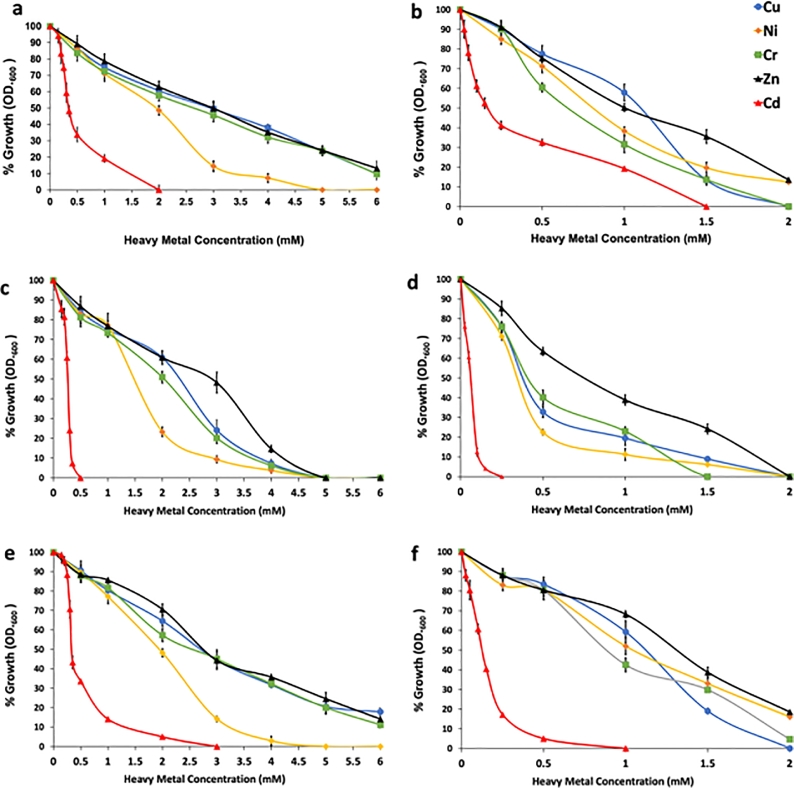
Examples of growth curves using LB and UM medium supplemented with escalated heavy metal concentrations; a) *Pseudomonas aeruginosa* (QZ9-LB), b) *Pseudomonas aeruginosa* (QZ9-UM), c) *Bacillus sp*. (QD1-LB), d) *Bacillus sp*. (QD1-UM), e) *Providencia rettgeri* (QZ2-LB), and f) *Providencia rettgeri* (QZ2-UM). Data were obtained from three replicate experiments. Error bars are standard deviations (SD).

The 11 isolates were used to explore their ability to grow and tolerate the toxicity exhibited by different heavy metals (Cr, Cu, Zn, Ni, and Cd) in two culture media: 1) LB, a rich medium, and 2) UM, containing fewer organic compounds. All 11 bacterial strains showed different growth profiles in both media for the studied range of concentrations (Fig. 1 & Fig 1S in Supplementary Material). The studied strains exhibited higher tolerance (MIC > 6 mM) to heavy metals when grown on LB-HM medium than when grown on UM-HM medium (MIC 3 mM). Cd exerted the highest toxicity to all studied strains with MIC up to 3 mM, 1.5 mM on LB-HM and UM-HM respectively.

### 3.3 Using PCA clustering for selection of bacterial strains

**Fig. 2:**
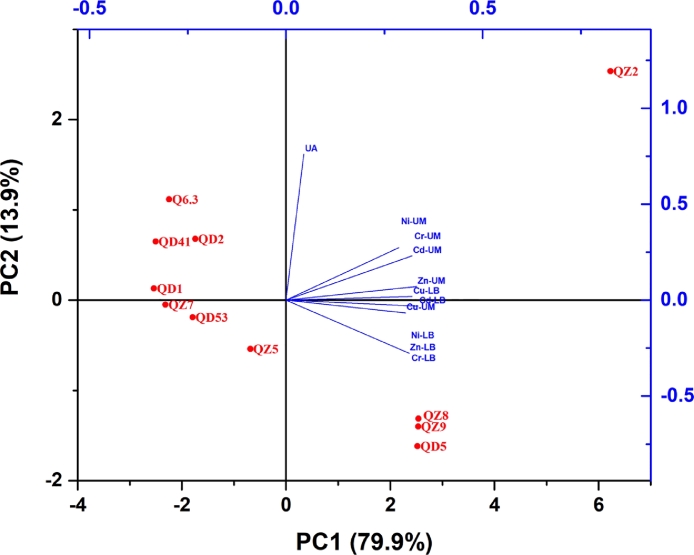
PCA Clustering of the studied bacterial strains showing three formed groups (I, II and II). The blue lines point to the factors responsible for the variations.

The MIC of heavy metals for the each of studied bacterial strains in both LB and UM (Figure 1 & Figure 2S) and production of urease enzyme (Table 2S) were used to perform Principal Component Analysis (PCA). Figure 2 indicates that PC1 and PC2 accounted for 79.9% and 13.9% variability, respectively. Hence, the first two components explained 93.8% of the variance of data, leading to a good discrimination of samples. Three groups could be obtained from PCA (Fig. 2), Group 1 contained seven bacterial strains (2 *Bacillus cereus* (QZ6 and Q6.3)*,* 2 *Bacillus subtilis* (QD2 and QD53), 2 *Bacillus licheniformis* (QD1 and QD41) and one *Stenotrophomonas sp.* (QZ5). Group II contained the three *Pseudomonas aeruginosa* strains (QZ8, QZ9 and QD5). Group III the contained *Providencia rettgeri* (QZ2).

Group 1 characterized with relatively lower tolerance to the studied heavy metals can be subdivided into two parts, part 1 containing (Q6.3, QD1, QD2 and Q41) is characterized by high production of urease activity, while part 2 containing (QD53, QZ7 and QZ5) is characterized with low urease activity. Group II is characterized by high tolerance to heavy metal and relatively low urease production. The *Providencia rettgeri* (QZ2*)* in group III is characterized with high tolerance to heavy metals as well as high production of urease. Based on the PCA results three bacterial strains (2 *Pseudomonas aeruginosa* (QZ9 & QD5) and *Providencia rettgeri* (QZ2) were selected for immobilization of heavy metals investigations.

### 3.4 Analysis of heavy metal removal

**Fig. 3:**
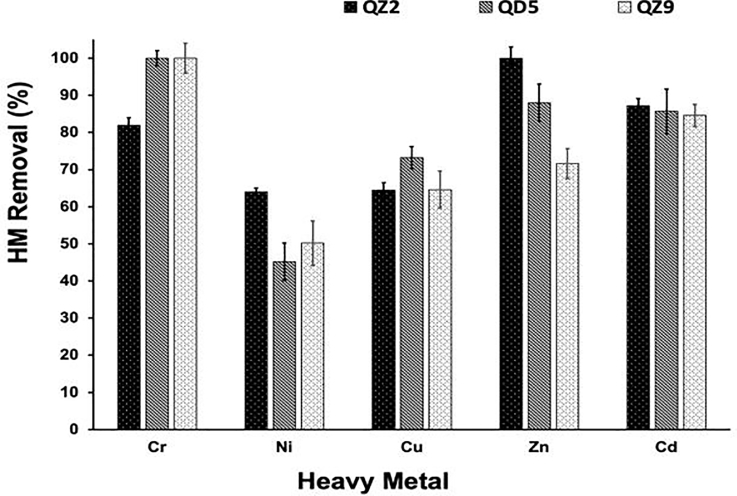
Total Removal efficiency (%) of heavy metals by the studied strains, *Providencia rettgeri* (QZ2) and *Pseudomonas aeruginosa* (QD5 & QZ9). Data were obtained from three replicate experiments. Error bars are standard deviations (SD).

The efficiency of Providencia rettgeri (QZ2) and Pseudomonas aeruginosa (QD5 and QZ9) to remove heavy metals was evaluated in the UM cultures supplemented with 0.1M of CaCl_2_ to induce calcium carbonate formation in the presence of 1 mM of each heavy metal. Total heavy metal removal efficiency and the proportions of heavy metal presented in the precipitated minerals and in washing solutions were determined by ICP-OES. The results shown in Fig. 3 indicate variable removal efficiencies depending on the heavy metals and the studied strain. The two Pseudomonas aeruginosa strains (QZ5 and QZ9) exhibited complete removal by biomineralization of Cr (100%) while the Providencia rettgeri (QZ2) strain showed 100% removal of Zn. The lowest removal efficiencies of (45 ± 5%) were recorded for Ni by Pseudomonas aeruginosa (QD5).

**Fig. 4:**
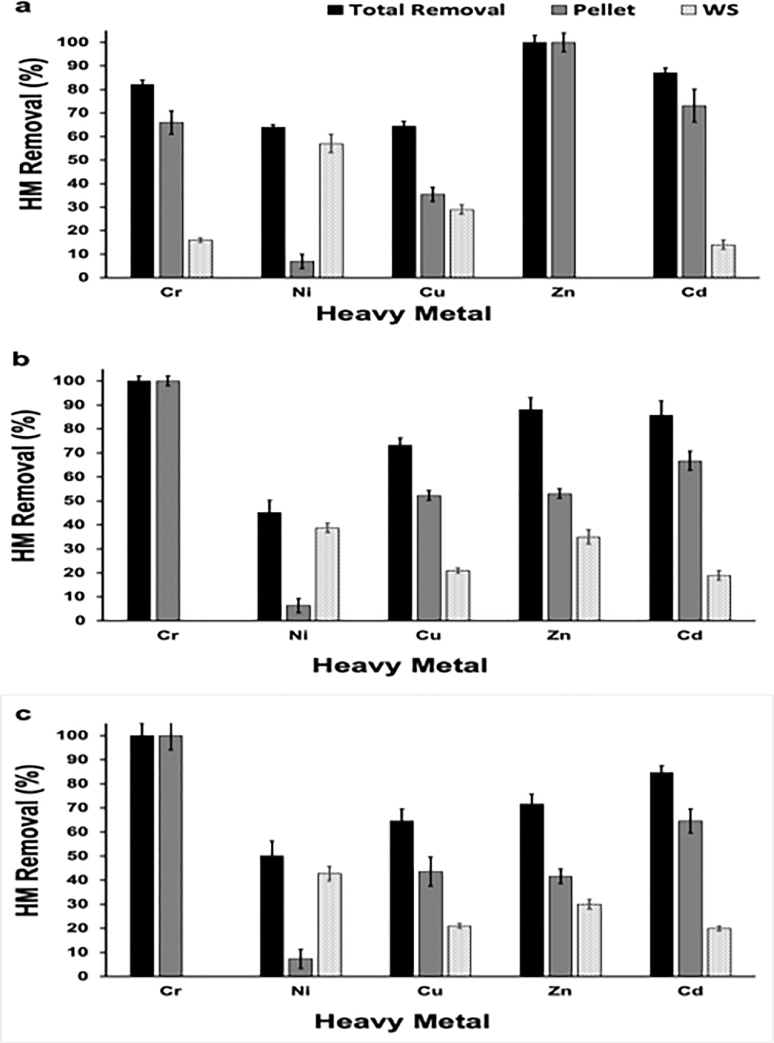
Proportions of heavy metals in precipitates (Pellet) and washing solutions (WS) recovered from the cultures of, a) *Providencia rettgeri* (QZ2), b) *Pseudomonas aeruginosa* (QD5) and c) *Pseudomonas aeruginosa* (QZ9). Data were obtained from three replicate experiments. Error bars are standard deviations (SD).

To have further insights on the fate of heavy metals, the proportions of heavy metals in the precipitated pellets and in washing solution was determined using ICP-OES. Results are shown in Figure 4. The lowest % of heavy metal removal was recorded with the minerals precipitated in the cultures supplemented with Ni (6-7%). While 39% - 57% of precipitated Ni was removed by washing. No heavy metals were detected in washing solutions in the conditions where 100% of the heavy metals were removed via bioprecipitation.

### 3.5 Characterization of the mineral precipitates

#### 3.5.1 Variability in the morphology and composition of the formed minerals in the presence of heavy metals using SEM mapping and EDS

**Fig. 5:**
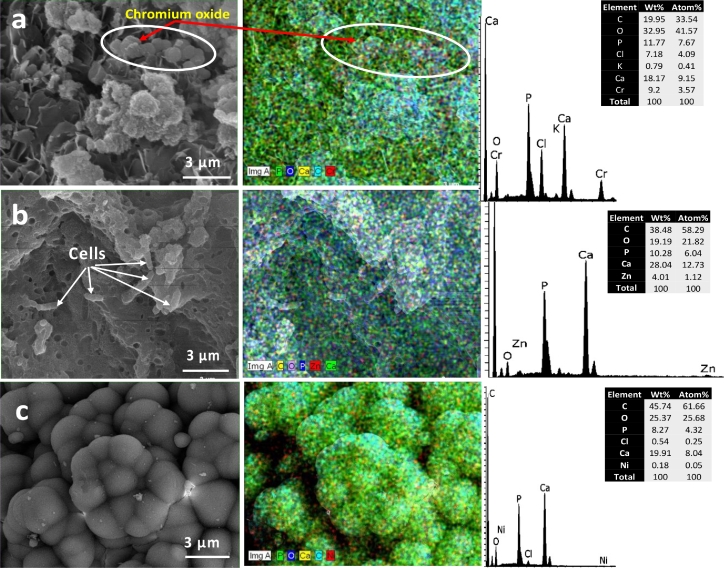
SEM images (left panel) and SEM mapping (center panel) of the minerals formed in *Providencia rettgeri* (QZ2) cultures: a) Chromium oxide formed in UM-Cr culture, b) Bacterial cells associated with formed minerals in UM-Zn culture, and c) Calcium phosphates formed in UM-Ni. EDS spectra (right panel) illustrate the elemental composition of the precipitates. The tables indicate the atom % of each element.

**Fig. 6:**
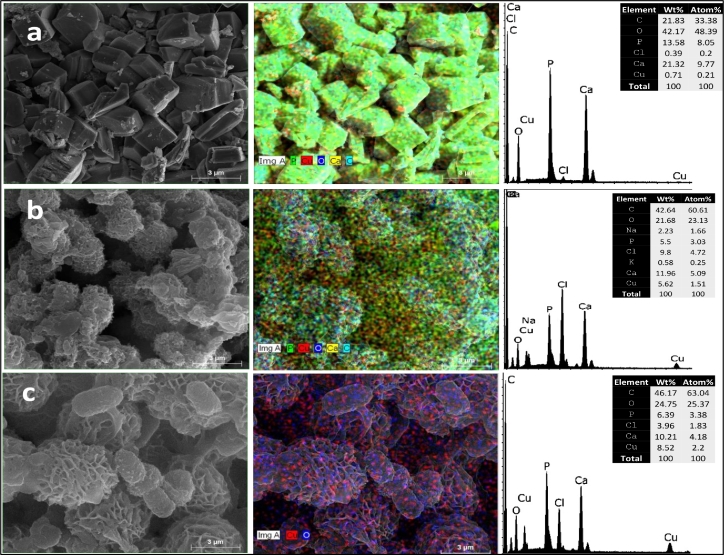
SEM images (left panel) and SEM mapping (center panel) of the minerals formed in *Pseudomonas aeruginosa* (QZ9) UM cultures in the presence of Cu. EDS (right panel) illustrate the elemental composition of the formed minerals. The tables indicate the atom % of each element.

The SEM analysis of the recovered minerals revealed high variability in their morphology and composition (Fig. 5, Fig. 6). The mineral phases and morphologies varied depending on the heavy metal added to the culture. In the cultures performed in UM media without heavy metals, both calcium carbonates and calcium phosphates were observed. The SEM/EDS analysis of the precipitates formed by *Providencia rettgeri* (QZ2) in the UM-Cr cultures indicated the presence of Cr (9.2 wt.%) in the form of spherical crystals (Fig 5a). The incorporation of Zn (up to 4.01 wt.%) was noted in precipitated minerals formed in QZ2-UM-Zn cultures (Fig 5b). The spherical calcium phosphate aggregates observed in the QZ2-UM-Ni cultures showed poor incorporation of Ni (0.18 wt. %) (Fig 5c). The precipitates formed in the *Pseudomonas aeruginosa* (QZ9) cultures supplemented with 1 mM Cu were characterized with different morphologies (Fig 6). The highest incorporation of Cu (8.52 wt.%) was observed with flower-like brushite crystals (Fig. 6c), whereas the lowest Cu occurrence (0.7 wt.%) was among the cubical calcite crystals (Fig. 6a). Moreover, Cu was spotted on the surface of bacterial cells (Fig 6b) indicating their possible adsorption capacity.

#### 3.5.2 Evaluation of the effect of heavy metals on the mineral composition using XRD analysis

**Fig. 7:**
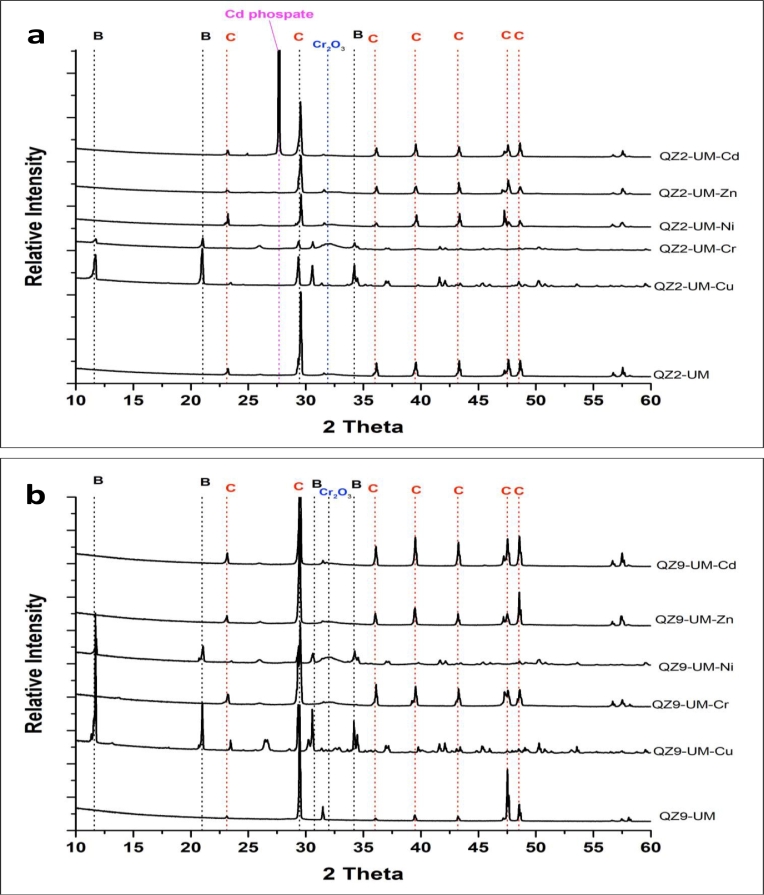
Illustrative XRD patterns of minerals recovered from the UM cultures of a) *Providencia rettgeri* (QZ2) and b) *Pseudomonas aeruginosa* (ZA9) supplemented with 1 mM of each studied heavy metal. B: Brushite, C: Calcite.

The XRD analysis indicated the formation of calcite (calcium carbonate) and brushite (calcium phosphates) at the studied conditions but in different proportions (Figure 7 and Table 1). In the cultures performed using the UM medium without heavy metals, the precipitation of calcium carbonates as a major mineral phase (>82%) and calcium phosphates as a minor phase (<18%) were observed. Cadmium phosphate (30% of total bulk) was only formed in the QZ2 cultures supplemented with 1mM of Cd (Fig. 7a), whereas chromium oxide formed in the precipitates obtained in the UM cultures in the presence of Cr (Fig. 7b).

## 4 Discussion

Eleven isolates were selected due to their potential for hydrocarbon degradation and/or mineral formation, as previously demonstrated in the laboratory (Al [[29], [30], [31], [33], [34], [35]]). All the studied strains in this study exhibited the capability to form minerals via MICP. They are used in this study to explore their ability to grow and tolerate the toxicity of different heavy metals (Cr, Cu, Zn, Ni, and Cd) in two growth media: (1) LB, a rich medium, and (2) UM, with fewer organic compounds. The LB and UM media were supplemented with one heavy metal in each liquid culture, at escalated concentrations from 0 to 10 mM. The growth of each strain was evaluated by an optical density approach. The evaluation of the growth of each bacterial strain allowed for estimation of the cell density, with viable and non-viable forms in the culture. The non-viable cells can also adsorb heavy metals [37]. All 11 bacterial strains showed different growth profiles in both media at the range of concentrations. This can be observed by comparing the tolerance of the strains in LB and UM media. Although the tolerance to several heavy metals was higher in LB than in UM, a threshold of almost 1 mM of each of Cr, Cu, Zn, and Ni and 0.5 mM of Cd were observed in LB and UM.

It has been theorized that the values of MIC vary depending on the type of culture medium [38]. Heavy metals may form a complex with components of bacterial growth media and thus remove them from the solution resulting in a reduction in their concentrations in the media [39]. In addition, the toxicity of metals may be reduced by common components of the medium nutrients. Leitao and Sa-Correia [40] demonstrated that the growth of *Pseudomonas aeruginosa* was improved in the presence of inhibitory concentrations of Cu with increased nutrient concentrations in the medium. As the bacterial cells would be exposed to lower concentrations of heavy metals than those reported, the interactions between components of the medium and the heavy metals would cause the MIC values to be overestimated [38]. Hence, the media constituents should be considered when studying the toxicity of metals to bacteria, otherwise, such studies may not reflect the actual situation in a natural environment.

Pseudomonas and *Providencia* strains exhibited the highest tolerance to all the tested heavy metals. This is in agreement with other recent works (i.e., [41], [42], [43]). By contrast, *Bacillus* and *Stenotrophomonas* strains showed lower tolerance to the studied heavy metals. In general, Zn, Cr, and Cu were the least toxic heavy metals whereas Ni and Cd exerted significantly higher toxicity levels (P-value < 0.001) with a MIC of 1.5 mM and 0.5 mM, respectively. Based on these results, the *Pseudomonas* and *Providencia* strains were selected to study the impact of heavy metals on the mineral formation and composition using a concentration of 1mM as the common minimum inhibitory threshold for Cd, Cr, Cu, Zn, and Ni.

The determination of the bacterial growth and urease activity was performed. Significant variations in the growth profiles (P value <0.001) were shown among the studied isolates. However, the highest growth was not necessarily associated with higher ureolytic activity in the medium. For example, the CFU of *Pseudomonas aeruginosa* (QD5) showed the highest biomass production (85 ± 4 × 10^6^ CFU/mL), but the corresponding AUA was the lowest (1.9 ± 0.2 AUA/mL). The highest specific production of AUA (7.7 ± 0.6 AUA/mL) and (6.1 ± 0.4 AUA/mL) was obtained by *Providencia rettgeri (*QZ2) and *Bacillus cereus* (Q6.3), respectively. Statistical analysis by ANOVA confirmed significant variations in the specific production of urease (P value <0.001). These results confirm obvious diversity among the 11 isolates. Although the *Bacillus* strains exhibited higher specific urease activity than the *Pseudomonas* strains, they showed lower tolerance to the studied heavy metals. For this reason, *Pseudomonas* and *Providencia* were selected for the investigation of mineral formation.

Calcium carbonates polymorphs (i.e., calcite) are reported to be precipitated by ureolytic bacteria via the MICP process [25, 44]. However, few reports are available on the formation of brushite (CaHPO_4_·2H_2_O) [45], but Heveran et al. [46] reported the formation of brushite by engineered ureolytic microorganisms. In our experiments, the XRD and SEM/EDS analyses confirmed the formation of calcite as a major mineral phase (> 80%) in the cultures performed in absence of any heavy metal, as well as in the cultures performed in the presence of Zn or Cd with minor amounts of brushite (<20%). However, the majority of formed minerals shifted from calcite to brushite in the UM cultures performed in presence of Cu and Ni. Brushite was formed as a major mineral phase (>61%) in the presence of Cu and Ni. Conversely, the formation of brushite was associated with significantly lower heavy metal removal efficiency of those heavy metals. Recent reports demonstrated that divalent heavy metals can interact with MICP [23, 47]. It also has been shown that heavy metals such as cadmium, cobalt, nickel, zinc may alter the calcite dissolution process [47]. The incorporation of these heavy metals results in inhibition or enhancement of the solubility of calcite, depending on the type of metals and their content [23]. The formation of chromium oxide (Cr_2_O_3_) in the *Pseudomonas aeruginosa* (QD5 & QZ9) UM-Cr cultures were combined with 100% of removal efficiency. This is consistent with the findings of Huang et al. [48], in which the formation of complex compounds (3CaO·Cr_2_O_3_·CaCO_3_·11H_2_O) was reported as a primary bioremediation mechanism to induce heavy metal ions to form insoluble precipitates.

Our results showed that the studied isolates were able to convert up to 100% of soluble Cr and Zn into insoluble minerals. Moreover, variable proportions of the heavy metals were transformed into insoluble forms via adsorption/desorption processes as demonstrated through their removal by washing solutions. These finding are in agreement with previous published reports (Aryal, 2020; Kaur et al., 2022). Using SEM/EDS mapping, the presence of Cd, Cr, Cu, Zn, and Ni in the precipitates formed by the selected bacteria was evidenced. Moreover, the remaining proportions of the partially removed heavy metals in both cell free supernatants and washing solutions were determined by ICP-OES. The remaining soluble and washed fractions indicate different removal behavior that may include adsorption-desorption processes [49].

## Conclusion

The immobilization of divalent heavy metals (Cd, Cr, Cu, Ni and Zn) based on microbially induced carbonate precipitation by ureolytic bacteria was investigated. The hydrocarbon-degrading ureolytic bacteria exhibited high tolerance to toxicity of heavy metals. Moreover, we demonstrated an outstanding performance in in the immobilization of heavy metals with removal efficiency reaching 100% of Cr by the *Pseudomonas* strains (QD5 & QZ9*).* Whereas the *Providencia rettgeri* strain (QZ2) showed 100% removal of Zn. The precipitated minerals shifted between calcium carbonate (calcite) and calcium phosphate (brushite) depending on the type of heavy metal. Our results demonstrated the potential of MICP implementation in the removal of heavy metals. MICP when combined with remediation of oil hydrocarbons should be considered as an effective bioremediation approach.

## Funding

This publication was made possible by PDRA Grant No. PDRA5-0425-19007 from the Qatar National Research Fund (a member of Qatar Foundation). The statements made herein are solely the responsibility of the authors.

## Declaration of Competing Interest

The authors declare that they have no known competing financial interests or personal relationships that could have appeared to influence the work reported in this paper

## Appendix Supplementary materials

Image, application 1
