# Supplementary material for: Immobilization of heavy metals by microbially induced carbonate precipitation using hydrocarbon-degrading ureolytic bacteria
Source: Biotechnol Rep (Amst). 2022 Jun 9;35:e00747. doi: 10.1016/j.btre.2022.e00747 (PMC9218142; doi:10.1016/j.btre.2022.e00747)
Supplement: Supplementary file 1 [file mmc1.docx]

**Insights into immobilization of heavy metals by microbially induced carbonate precipitation (MICP) using ureolytic-hydrocarbon degrading bacteria**

**Supplementary Data**

**
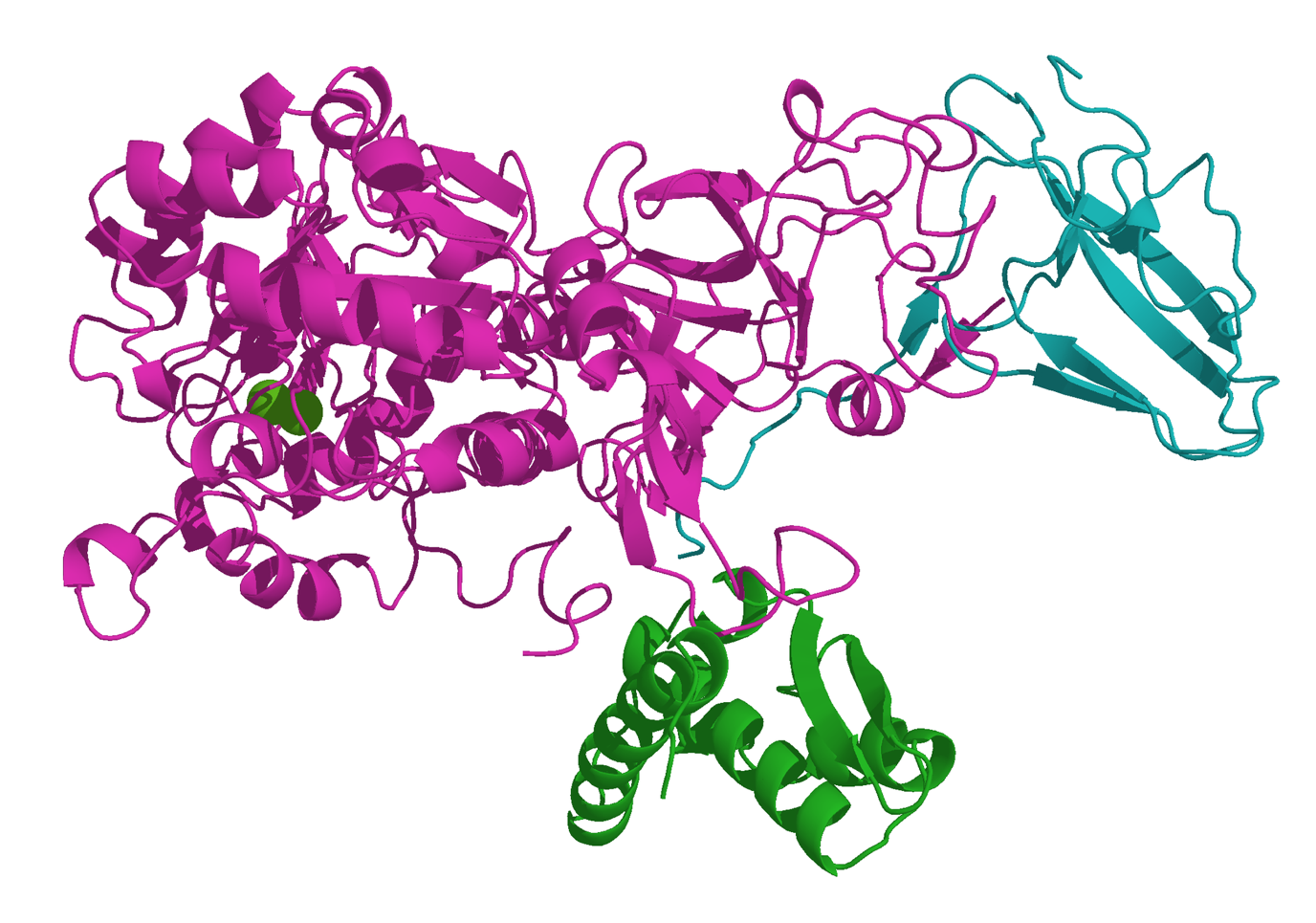
**Figure 1S: *C*rystal structure *Illustration* of urease *enzyme* from Klebsiella aerogenes as reported by (Jabri et al., 1995).

Table 1S: Bacterial strains used in the study.

| No. | Code | Strain Name | Isolation site | GenBank Accession No. | Reference: |
| --- | --- | --- | --- | --- | --- |
|  | QZ8 | *Pseudomonas aeruginosa* | Automotive workshop | CP015377.1 | (Al Disi et al., 2017b) |
|  | QZ9 | *Pseudomonas aeruginosa* | Automotive workshop | JF919950.1 | (Al Disi et al., 2017b) |
|  | QD5 | *Pseudomonas aeruginosa* | Dukhan Dumping site | KY040017.1 | (Al-Kaabi et al., 2018) |
|  | QZ7 | *Bacillus cereus* | Dukhan | DQ120941.1 | (Al-Kaabi et al., 2018) |
|  | Q6.3 | *Bacillus cereus* | Dukhan | MG751339 | (Bibi et al., 2018) |
|  | QD1 | *Bacillus licheniformis* | AlZubara | LN995452.1 | (Al-Kaabi et al., 2020) |
|  | QD41 | *Bacillus licheniformis* | Dohat Faishakh Sabkha | KY363571 | (Al Disi et al., 2017a) |
|  | QD2 | *Bacillus subtilis* | Dukhan Dumping site | MH071337.1 | (Al-Kaabi et al., 2018) |
|  | QD53 | *Bacillus subtilis* | Dukhan | MALDI-TOF MS Score (1.95) | (Alsayegh et al. 2021) |
|  | QZ2 | *Providencia rettgeri* | AlZubara | CP027418.1 | (Al-Kaabi et al., 2020) |
|  | QZ5 | *Stenotrophomonas sp.* | GTL water | KX036541.1 | (Surkatti et al., 2021) |

Table 2S: Arbitrary urease activity (AUA) and its specific production by each bacterial isolate

| Isolate | CFU (10^6^ CFU/mL) | Arbitrary urease activity (AUA/ mL) | Specific production (AUA/10^7^ CFU) |
| --- | --- | --- | --- |
| QZ8 | 65 ± 3 | 17.3 ± 6 | 2.6 ± 0.8 |
| QZ9 | 75 ± 5 | 18.6 ± 8 | 2.4 ± 0.6 |
| QD5 | 85 ± 4 | 16.5 ± 5 | 1.9 ± 0.2 |
| QZ7 | 33 ± 3 | 11.7 ± 7 | 3.4 ± 0.7 |
| Q6.3 | 17 ± 2 | 11.0 ± 9 | 6.1 ± 0.4 |
| QD1 | 17 ± 1 | 6.5 ± 3 | 3.7 ± 0.3 |
| QD41 | 37 ± 4 | 19.1 ± 14 | 4.9 ± 0.8 |
| QD2 | 39 ± 3 | 18.2 ± 10 | 4.5 ± 0.3 |
| QD53 | 33 ± 1 | 8.4 ± 2 | 2.5 ± 0.3 |
| QZ2 | 29 ± 2 | 23.1 ± 11 | 7.7 ± 0.6 |
| QZ5 | 31 ± 3 | 13.7 ± 9 | 4.2 ± 0.6 |


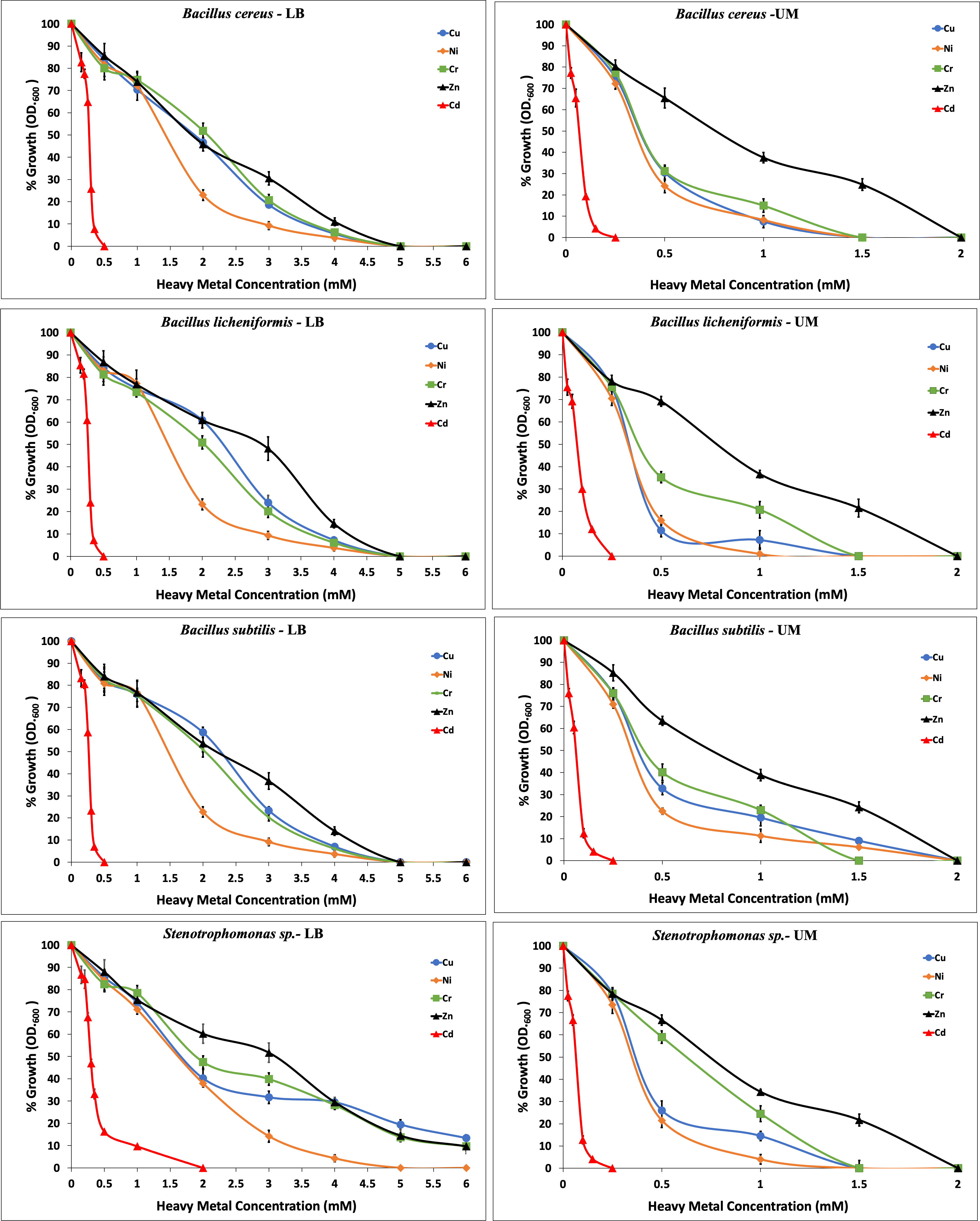


Figure 2S: Examples of growth curves of the studied bacterial strains using LB (right) and UM medium (left) supplemented with different concentrations of heavy metals (0-10 mM)

# References

Al Disi, Z. A., Jaoua, S., Bontognali, T. R., Attia, E. S., Al-Kuwari, H. A., & Zouari, N. (2017a). Evidence of a Role for Aerobic Bacteria in High Magnesium Carbonate Formation in the Evaporitic Environment of Dohat Faishakh Sabkha in Qatar. *Frontiers in Environmental Science, 5*, doi.org/10.3389/fenvs.2017.00001.

Al Disi, Z., Al-Thani, D., Jaoua, S., Al-Meer, S., & Zouari, N. (2017b). Considering the Specific Impact of Harsh Conditions and Oil Weathering on Diversity, Adaptation, and Activity of Hydrocarbon-Degrading Bacteria in Strategies of Bioremediation of Harsh Oily-Polluted Soils. *BioMed Research International, 2017*, 11; https://doi.org/10.1155/2017/8649350.

Al-Kaabi, N., Al-Ghouti, M. A., Oualha, M., Mohammad, M. Y., Al-Naemi, A., Sølling, T. I., . . . Zouari, N. (2018). A MALDI-TOF study of bio-remediation in highly weathered oil contaminated soils. *Journal of Petroleum Science and Engineering, 168*(2018), 569-576. https://doi.org/10.1016/j.petrol.2018.05.013.

Alsayegh, S. Y., Al Disi, Z., A.Al-Ghouti, M., & Zouar, N. (2021). Evaluation by MALDI-TOF MS and PCA of the diversity of biosurfactants and their producing bacteria, as adaption to weathered oil components. *Biotechnology Reports, 31*, e00660.

Bibi, S., Oualha, M., Ashfaq, M. Y., Suleiman, M. T., & Zouari, N. (2018). Isolation, differentiation and biodiversity of ureolytic bacteria of Qatari. *RSC Advances, 8*, 5854–5863.

Surkatti, R., Disi, Z. A., El-Naas, M. H., Zouari, N., Loosdrecht, M. V., & Onwusogh, U. (2021). Isolation and Identification of Organics-Degrading Bacteria From Gas-to-Liquid Process Water. *Frontiers in Bioengineering and Biotechnology, 8*, https://doi.org/10.3389/fbioe.2020.603305.

Jabri, E., Carr, M. B., Hausinger, R. P., & Karplus, P. A. (1995). The crystal structure of urease from Klebsiella aerogenes. *Science, 19*(268 (5213)), 998-1004.
